# Intravenous paracetamol for fever control in acute brain-injured patients: cerebral and hemodynamic effects

**DOI:** 10.1186/cc12267

**Published:** 2013-03-19

**Authors:** E Picetti, I Rossi, P Ceccarelli, S Risolo, P Schiavi, V Donelli, A Crocamo, M Antonini, M Caspani

**Affiliations:** 1 I Servizio Anestesia e Rianimazione, Azienda Ospedaliero-Universitaria di Parma, Italy; 2 Neurochirurgia e Neurotraumatologia, Azienda Ospedaliero-Universitaria di Parma, Italy

## Introduction

Fever is a dangerous secondary insult for the injured brain [1]. We investigated the cerebral and hemodynamic effects of intravenous (i.v.) paracetamol administration for the control of fever in neurointensive care unit (NICU) patients.

## Methods

The i.v. paracetamol (1 g in 15 minutes) was administered to NICU patients with a body temperature (Temp.) >37.5°C. Its effects on mean arterial pressure (MAP), heart rate (HR), intracranial pressure (ICP), cerebral perfusion pressure (CPP), jugular venous oxygen saturation (SjVO_2_) and Temp. were recorded at the start of paracetamol infusion (T0) and after 30 (T30), 60 (T60) and 120 (T120) minutes. Interventions for the maintenance of CPP >60 mmHg or ICP <20 mmHg were recorded.

## Results

**Figure 1:**
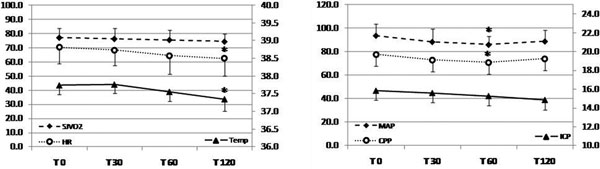
*P <0.05 versus T0.

Fifteen NICU patients (nine subarachnoid hemorrhage, five traumatic brain injury, mean age 54.9 ± 16.8, seven (50%) males, median GCS 7) were prospectively studied. We analyzed the administration of one dose of paracetamol for each patient (total 14 cases). After infusion of paracetamol we found a decrease of Temp. (from 37.8 ± 0.3 to 37.4 ± 0.4°C, *P <*0.001), MAP (from 94.7 ± 9.9 to 86.1 ± 6.7 mmHg, *P *= 0.008), CPP (from 79.6 ± 13.1 to 70.8 ± 7.6 mmHg, *P *= 0.011) and HR (from 71.5 ± 14.9 to 63.8 ± 16.3 bpm, *P <*0.001) with respect to the starting value (ANOVA for repeated measures), whereas ICP and SjVO_2 _remained unchanged (Figure 1). In five cases norepinephrine infusion was started for CPP <60 mmHg. In another two cases, for the same reason, the norepinephrine dosage was augmented. The proportion of patients who had infusion of norepinephrine increased from 42.8% at T0 to 78.6% at T120 (*P *= 0.02, chi-square for trends).

## Conclusion

Use of i.v. paracetamol is effective in the maintenance of normothermia in acute brain-injured patients. However, adverse hemodynamic effects, which could represent a secondary insult for the injured brain, must be rapidly recognized and treated.
